# Body composition radiomics integrated with machine learning to predict prognosis in advanced NSCLC treated with first-line immunochemotherapy and concurrent radiotherapy

**DOI:** 10.1186/s12890-026-04405-w

**Published:** 2026-06-19

**Authors:** Wen Xu, Yongze Yu, GongHua Dai, Jian He, Xin Li, Li Zhang, Xuejiao Zeng, Lungui Hu, Jinyuan Zhang, Qiyu Fang, Lijun Liao

**Affiliations:** 1 Department of Medical Imaging, Shanghai East Hospital (East Hospital Affiliated with Tongji University), No. 150 Jimo Road, Pudong New Area, Shanghai, 200120 P. R. China; 2 Department of Pain Management, Shanghai East Hospital, School of Medicine, Tongji University, Shanghai, 200120 China; 3 School of Medical and Information Engineering, Gannan Medical University, Ganzhou, 341000 China; 4 Department of Medical Oncology, Shanghai Pulmonary Hospital, School of Medicine, Tongji University Medical School Cancer Institute, Tongji University, Shanghai, 200433 China

**Keywords:** Non-small cell lung cancer, CT, Skeletal muscle, Adipose tissue, Radiomics, Machine learning

## Abstract

**Objective:**

Adipose tissue is a highly heterogeneous and plastic endocrine and immune organ whose characteristics have been linked to prognosis in NSCLC patients receiving First-Line Immunochemotherapy and Concurrent Radiotherapy. This study aimed to investigate whether baseline body composition and radiomics features could serve as prognostic predictors in this patient population.

**Method:**

This retrospective study involved the collection of data from 87 patients receiving chemotherapy in conjunction with immunotherapy. Radiomic features were extracted from skeletal muscle and adipose tissue at the L3 level of the lumbar spine. The most reliable radiomics features were selected to develop six machine learning classifier models. Patients were stratified into high-risk and low-risk groups based on the determination of an optimal threshold. Subsequently, TATI(Total adipose tissue index) and radiomics features were integrated to develop comprehensive predictive model, and the model’s performance was assessed using ROC and DCA.

**Results:**

The results indicated that the AUC for the combined model was 0.857 (95% CI: 0.765–0.949) in the train cohort and 0.834 (95% CI: 0.740–0.934) in the test cohort. Subsequently, the risk stratification analysis revealed that PFS was significantly shorter in high-risk patients compared to low-risk patients. Our model displayed enhanced predictive accuracy and greater net benefit.

**Conclusion:**

The research findings indicate that radiomics features derived from body composition hold potential for predicting prognosis in patients with non-small cell lung cancer. Our model may identify high-risk cohorts, thereby assisting in the identification of patients likely to benefit from concurrent radiotherapy through optimised treatment regimens.

**Supplementary Information:**

The online version contains supplementary material available at https://doi.org/10.1186/s12890-026-04405-w.

## Introduction

As reported in the World Health Organization’s (WHO) 2022 report, lung cancer is the most common cancer worldwide, accounting for 12.4% of all cancer cases and the highest mortality rate, with 18.7% of all cancer-related deaths [1]. Non-small cell lung cancer (NSCLC) accounts for approximately 85% of all lung cancer cases. At diagnosis, 30% of patients present with locally advanced disease (stage III), while 25–30% have unresectable locally advanced NSCLC (LA-NSCLC) [2, 3]. With advances in therapeutic techniques, concurrent radiotherapy (CRT) has become a cornerstone of treatment for unresectable locally advanced NSCLC, providing significant and sustained survival benefits [4]. Consequently, there is an urgent need for effective prognostic tools to improve patient outcomes and minimize treatment-related toxicity in this patient population.

Medical imaging is the standard of care for early detection, diagnosis, treatment planning, surveillance, and image-guided interventions in lung cancer patients. In conventional radiology, features such as shape, position, volume, density, foliation, and standardized uptake values (SUV), derived from radiographs, CT, MRI, and PET/CT imaging, are commonly used to assess lung cancer [5]. However, these features are typically evaluated visually by radiologists, introducing subjectivity and limiting their accuracy. The emerging field of imaging genomics, which converts medical images into quantifiable data, offers non-invasive biomarkers for diagnosis, prognosis, monitoring treatment response, and tracking disease progression over time [6].

While body mass index (BMI) is a widely used indicator for assessing obesity, the “obesity paradox” highlights its limitations in evaluating body composition. Significant variations in body composition exist across individuals, influenced by factors such as gender and age. Thus, two patients with the same BMI may have markedly different body compositions and clinical outcomes. Moreover, the effect of these variations on the prognosis of NSCLC patients is not fully understood [7, 8]. The distribution of muscle and adipose tissue has been identified as a critical factor in cancer outcomes, affecting postoperative complications, chemotherapy-related toxicity, and overall survival [9]. Adipose tissue, one of the largest endocrine organs, secretes several adipokines, including leptin, lipocalin, and interleukin-6 (IL-6), which have been shown to serve as prognostic markers in various cancers [10]. The interaction between cancer and adipose tissue plays a crucial role in mediating adipose dysfunction, thereby promoting cancer progression [11]. The development of body composition assessment methods using routine clinical imaging has revolutionized this field. Growing evidence suggests that quantifying muscle and adipose tissue via clinically acquired imaging can help personalize treatment plans and optimize outcomes for cancer patients [12].

This study aimed to explore body composition radiomics with the aim of developing a prognostic model for advanced NSCLC patients receiving First-Line Immunochemotherapy and Concurrent Radiotherapy.Ultimately, this model provides clinicians with a tool capable of capturing heterogeneity to identify advanced NSCLC patients most likely to benefit from First-Line Immunochemotherapy and Concurrent Radiotherapy.

## Method

### Patients

This retrospective study reviewed electronic medical records of patients with pathologically diagnosed advanced non-small cell lung cancer (NSCLC), as per the International Association for the Study of Lung Cancer (IASLC) guidelines (8th edition). These patients were treated with first-line immunotherapy (PD-1 inhibitor) and Concurrent Radiotherapy between March 2017 and March 2019 at Shanghai Pulmonary Hospital.

This retrospective study conducted a retrospective analysis of electronic medical records for patients with pathologically confirmed advanced non-small cell lung cancer (NSCLC) at Shanghai Pulmonary Hospital between March 2017 and March 2019. These patients had previously received first-line PD-1/PD-L1 immune checkpoint inhibitor (ICI) therapy in combination with chemotherapy.

In this study, patients with locally advanced NSCLC were defined as those who could not undergo complete resection or radical concurrent/sequential radiotherapy due to metastatic lesions in different lung lobes on the same side.

Inclusion criteria were as follows:

- (a) Wild-type driver genes, such as epidermal growth factor receptor (EGFR), anaplastic lymphoma kinase (ALK), or ROS proto-oncogene 1 (ROS1);
- (b) Eastern Cooperative Oncology Group Performance Status (ECOG PS) 0–2.

Exclusion criteria were:

c. (a) Absence of assessable CT scans with coverage of L3 within one month prior to the initiation of chemo-immunotherapy;
d. (b) Patients who received CTLA-4 inhibitors or other experimental compounds;
e. (c) Diabetic adults or patients undergoing exercise programs, weight loss programs, fecal transplants, or taking medications like metformin or MEK inhibitors, as these could affect body composition;
f. (d) Insufficient clinical or follow-up information;
g. (e) Lumbar spine surgery that might influence adipose or muscle evaluation.

This study was approved by the Ethics Committee of Shanghai Pulmonary Hospital (No.K22-241). As a retrospective study, the requirement for signed informed consent was waived.

### Clinical endpoint

The primary objective of this study was to evaluate durable clinical benefit, defined as progression-free survival (PFS). PFS was assessed using the criteria established by RECIST 1.1 [13], covering the interval from the start of the first immunotherapy cycle until either disease progression, death, or the last follow-up visit. We specifically aimed to assess the role of radiomics features in predicting PFS at 12 months. Patients with a PFS of 12 months or more who exhibited durable clinical benefit were classified as responders, while the remaining patients were categorized as non-responders.

### Image acquisition, segmentation and radiomics feature extraction

**Fig. 1:**
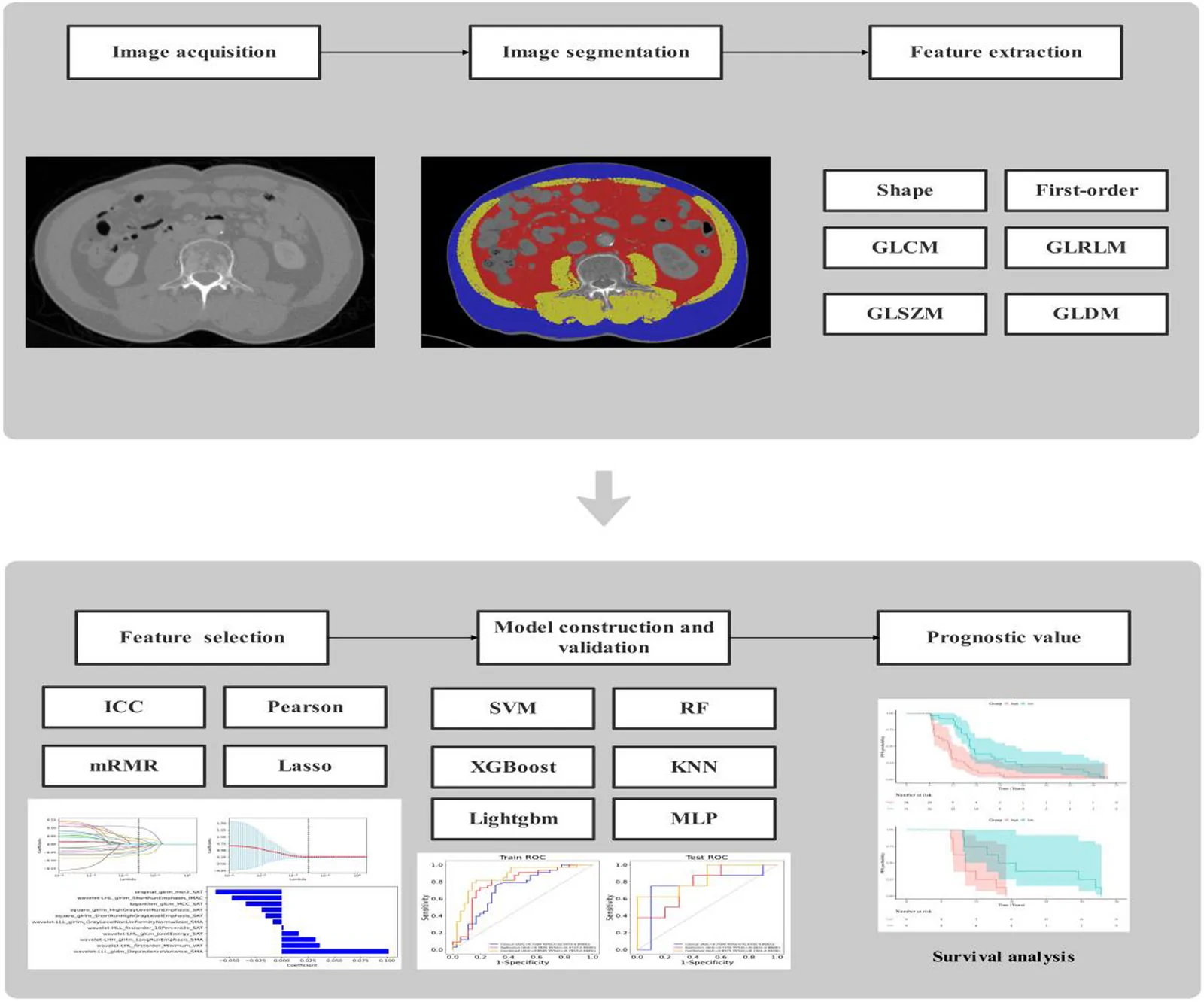
Workflow for screening radiomics features

The workflow is shown in Fig. 1. All patients underwent a computed tomography (CT) scan within one month prior to immunotherapy. A spiral CT scanner (uCT760, United Imaging and Brilliance iCT, Philips) was used for imaging. Segmentation of skeletal muscle and adipose tissue at the L3 level was performed by an experienced radiologist using the open-source software 3D Slicer (version 5.0.2, www.slicer.org).

The segmentation was based on Hounsfield unit (HU) thresholds: skeletal muscle was thresholded at -29 to 150 HU, subcutaneous adipose tissue and intramuscular adipose tissue at -190 to -30 HU, and visceral adipose tissue at -150 to -50 HU [14]. Manual adjustments were made by the radiologist as needed.The Skeletal Muscle Index (SMI) was calculated as the skeletal muscle cross-sectional area (SMA) divided by height squared (cm²/m²) [15]. The following parameters were also calculated: subcutaneous fat area (SAT), visceral fat area (VAT), intramuscular fat area (IMAT), intramuscular fat index (IFI, cm²/m² = IMAT / height²), total adipose tissue index (TATI, cm²/m² = (SAT + VAT) / height²), visceral fat index (VFI, cm²/m²= VAT / height²), subcutaneous fat index (SFI, cm²/m²= SAT / height²), skeletal muscle cross-sectional area (SMA), skeletal muscle density (SMD), and the visceral-to-subcutaneous fat ratio (VSR)was calculated as VAT / SAT [16–18].

To minimize bias from data heterogeneity, image preprocessing was performed before feature extraction. This involved resampling the images to 1 × 1 × 1 mm³, discretizing the grey values with 25 bin widths, and normalizing the images to account for equipment-related differences. Radiomics features were then extracted using Pyradiomics (version 3.0.1, https://pyradiomics.

A total of 1223 radiomics features were extracted from each region, including 14 shape features, 234 first-order features, 312 grey level co-occurrence matrix (GLCM) features, 208 grey level run length matrix (GLRLM) features, and 208 grey level size zone matrix (GLSZM) features. Additional features included 182 grey level dependence matrix (GLDM) features, 65 neighbourhood grey-tone difference matrix (NGTDM) features, and 182 grey level zone matrix (GLZM) features, resulting in a total of 4892 features.

To assess the robustness of the extracted features, 40 patients were randomly selected and re-segmented by a second radiologist to evaluate inter-observer reproducibility.

### Radiomics feature selection and radiomics model construction

First, the repeatability (inter-observer agreement) of the features extracted from the regions of interest (ROI) was evaluated by calculating the intraclass correlation coefficient (ICC). Features with an ICC > 0.9 were considered highly stable and repeatable, and were retained for further analysis. Patients were randomly assigned to the train and test cohort in an 8:2 ratio. The train cohort was standardized, and these standardized parameters were subsequently applied to the test cohort.

To ensure optimal model performance, we employed several data reduction techniques to eliminate redundant and irrelevant features. These included: (1) a two-sample t-test, (2) Pearson correlation analysis (removing features with correlation *r* > 0.9), (3) Minimum Redundancy Maximum Relevance (mRMR) analysis to select the top 20 most relevant features from the radiomics data. Finally, 10-fold cross-validation with Lasso regression was used to further refine the feature cohort.

The radiomics score (Radscore) for each sample is calculated by integrating selected radiomic features using Lasso regression, represented as:

$$\:\mathrm{R}\mathrm{a}\mathrm{d}\mathrm{s}\mathrm{c}\mathrm{o}\mathrm{r}\mathrm{e}=\mathrm{i}\mathrm{n}\mathrm{t}\mathrm{e}\mathrm{r}\mathrm{c}\mathrm{e}\mathrm{p}\mathrm{t}\hspace{0.17em}+\hspace{0.17em}{\upbeta\:}\mathrm{i}\times\:\mathrm{X}\mathrm{i}$$

where β denotes the coefficient, X represents the feature, and ii indicates the ordinal number. Based on the Radscore, we developed a radiomics model utilizing six distinct machine learning classifiers: Random Forest (RF), Logistic Regression (LR), eXtreme Gradient Boosting (XGBoost), Support Vector Machines (SVM), Light Gradient Boosting Machine (LightGBM), and K-Nearest Neighbors (KNN). Model hyperparameters were optimized through a grid search embedded within a 10-fold cross-validation framework. The key parameters explored for each algorithm are summarized in Supplementary Table 1. These parameters represent the set that maximized the average cross-validation AUC score, ensuring a balance between model performance and generalizability.To ensure the robustness of the model, five-fold cross-validation was implemented.The model with the highest AUC is considered the optimal radiomics model.

### Combined model

These parameters were analyzed using both univariate and multivariate Cox regression models. Subsequently, the TATI and radiomics features were combined to construct a comprehensive predictive model. To assess the comparability of the clinical model, radiomics model, and clinical-radiomics model, the best-performing machine learning classifiers from the radiomics model were applied to validate the clinical model.

### Statistical analysis

This study utilized Python (version 3.6, www.python.org) and R (version 4.2.1) for statistical analysis. The chi-square test was applied to compare qualitative data, while the t-test was used for comparing normally distributed quantitative data. The model’s predictive performance was evaluated using the receiver operating characteristic (ROC) curve, which included calculating the area under the curve (AUC) along with its 95% confidence interval (CI), as well as accuracy, sensitivity, and specificity.

## Results

### Patient

**Fig. 2:**
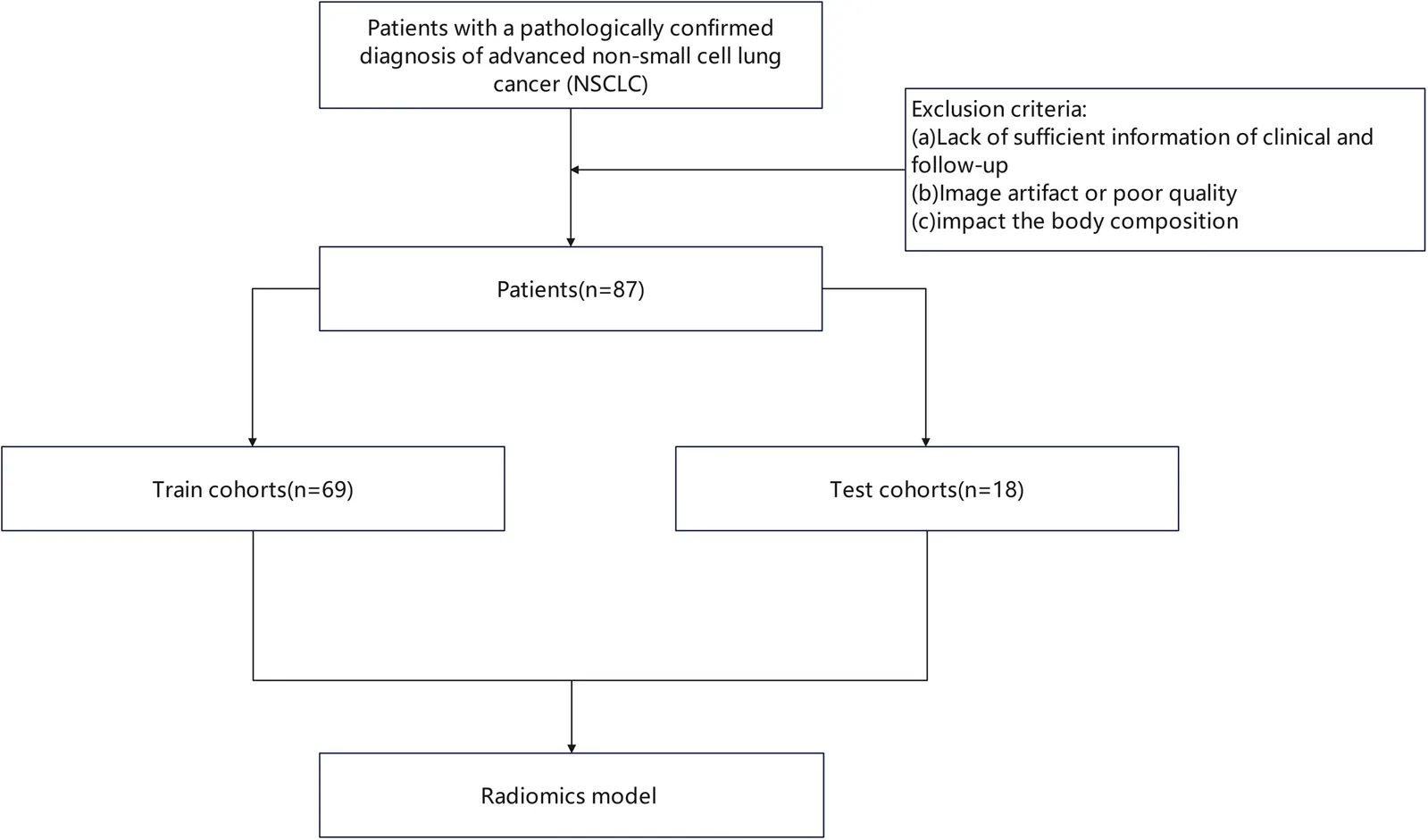
The flow chart for patient screening

The flowchart for patient screening is shown Fig. 2. A total of 108 patients with advanced-stage III and IV non-small cell lung cancer were retrospectively identified from the Shanghai Pulmonary Hospital records between 2017 and 2019. Of these, 87 patients met the inclusion criteria. The participants were then divided into a train cohort (*N* = 80) and a test cohort (*N* = 17) in an 8:2 ratio. The demographic and clinical characteristics of the patients are presented in Table 1, with no significant differences observed between the train and test cohort.

**Table 1:** Patients baseline characteristics

Variables	Total(*n* = 87)	train(*n* = 69)	test(*n* = 18)	*P*
Age, Mean ± SD	60.84 ± 8.05	60.80 ± 8.45	61.00 ± 6.53	0.925
BMI, Mean ± SD	23.29 ± 3.24	23.37 ± 3.33	22.96 ± 2.95	0.637
SMG, Mean ± SD	1760.80 ± 529.77	1784.48 ± 518.76	1670.00 ± 576.49	0.417
TFA, Mean ± SD	255.89 ± 116.63	253.02 ± 121.54	266.89 ± 97.82	0.656
TATI, Mean ± SD	91.18 ± 40.33	89.84 ± 41.86	96.32 ± 34.41	0.547
Haemoglobin, Mean ± SD	133.69 ± 15.03	133.61 ± 15.62	134.00 ± 12.95	0.922
Platelet, Mean ± SD	262.60 ± 74.22	259.52 ± 73.02	274.39 ± 79.70	0.452
SAT, Median (Q₁,Q₃)	122.60(88.19,146.30)	122.60(87.60,149.60)	122.95(91.74,143.28)	0.962
VAT, Median (Q₁,Q₃)	116.90(51.41,160.70)	108.90(48.74,161.80)	129.85(85.94,156.15)	0.285
IMAT, Median (Q₁,Q₃)	13.22(9.74,18.81)	12.90(9.30,18.78)	14.82(12.30,18.44)	0.260
SMD, Median (Q₁,Q₃)	39.10(33.56,42.97)	39.52(35.79,42.93)	37.52(29.59,44.65)	0.489
VSR, Median (Q₁,Q₃)	0.84(0.57,1.22)	0.76(0.57,1.20)	1.07(0.79,1.33)	0.117
SFI, Median (Q₁,Q₃)	42.82(33.48,55.29)	42.50(33.62,55.21)	44.40(33.41,54.93)	0.879
VFI, Median (Q₁,Q₃)	39.15(19.77,56.00)	37.68(17.87,54.80)	50.63(32.38,56.41)	0.222
IFI, Median (Q₁,Q₃)	4.68(3.49,6.60)	4.53(3.37,6.54)	5.44(4.52,6.67)	0.238
SMI, Median (Q₁,Q₃)	45.99(40.01,50.82)	46.45(39.95,50.99)	41.71(40.82,49.92)	0.679
Neutrophils, Median (Q₁,Q₃)	5.19(4.52,6.62)	5.24(4.52,6.66)	5.04(4.53,5.78)	0.710
Lymphocytes, Median (Q₁,Q₃)	1.77(1.40,2.25)	1.74(1.40,2.25)	1.86(1.67,2.23)	0.414
Gender, n(%)				1.000
female	12(13.79)	10(14.49)	2(11.11)	
male	75(86.21)	59(85.51)	16(88.89)	
Path, n(%)				0.226
LUSC	35(40.23)	30(43.48)	5(27.78)	
Non-LUCS	52(59.77)	39(56.52)	13(72.22)	
Transfer, n(%)				0.175
No	23(26.44)	21(30.43)	2(11.11)	
Yes	64(73.56)	48(69.57)	16(88.89)	
Stage, n(%)				1.000
Ⅲ	12 (13.79)	2 (11.11)	10 (14.49)	
Ⅳ	75 (86.21)	16 (88.89)	59 (85.51)	

*SMG* Skeletal Muscle Gauge, *TFA *total abdominal fat are, *TAFI *Total Fat Tissue Inde, *SAT *Subcutaneous adipose tissu, *VAT *Visceral adipose tissue, *IMAT *Intramuscular adipose tissu, *SMD *Skeletal muscle density, *VSR* Visceral/subcutaneous fat ratio, *SFI *Subcutaneous Fat Index, *VFI* Visceral Fat Index, *SMI* Skeletal Muscle Index, *LUCS* Lung squamous cell carcinoma

### Radiomics model

**Fig. 3:**
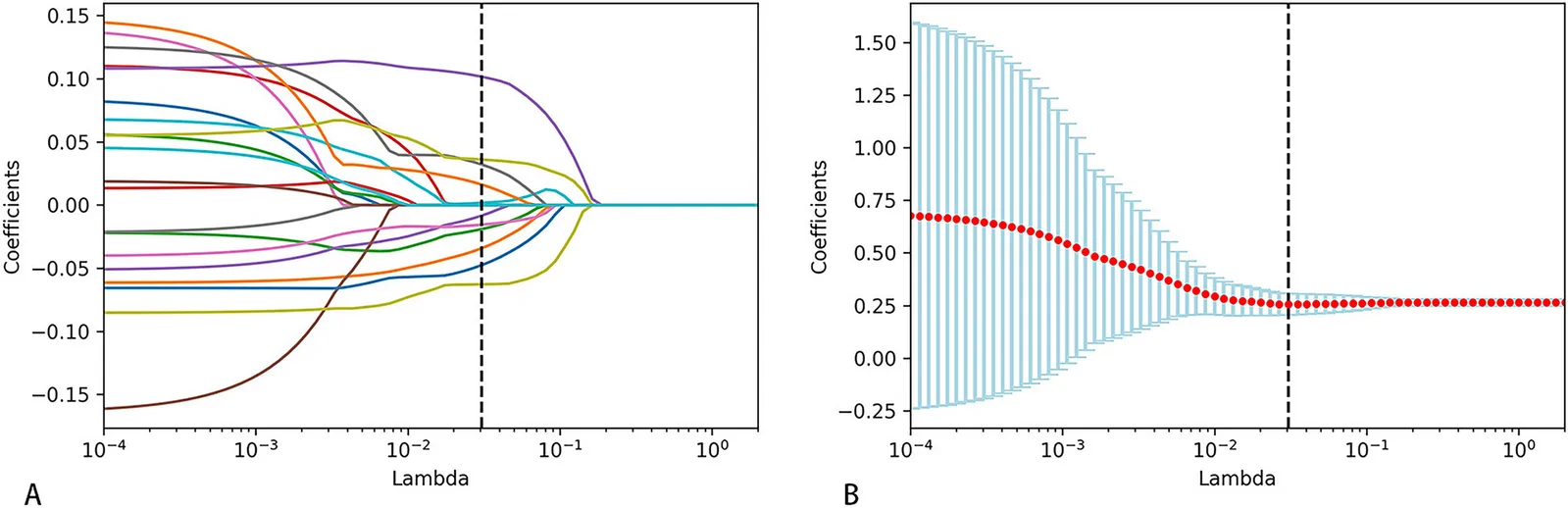
The figure illustrates the process of radiomics feature selection. (**A**) a The feature selection of LASSO regression is based on the minimum binomial deviation of the model plus one standard error to review features with non-zero coefficients. (**B**)b The 10-fold cross-validation process for LASSO regression feature selection

The radiomics features demonstrated an ICC > 0.9, and 170 radiomics features were identified as statistically significant in the train cohort. Subsequently, 83 redundant features with a Pearson correlation coefficient *r* > 0.9 were excluded, leaving 87 radiomics features for further analysis. The 20 most relevant radiomics features were selected using mRMR, and 11 features were chosen through a 10-fold cross-validation process applied to the Lasso regression method. These 11 features were assigned the following weights: 3 features derived from skeletal muscle, 6 features from subcutaneous adipose tissue, one from visceral adipose tissue, and one from intramuscular adipose tissue. For a detailed illustration, please refer to Fig. 3. Table 2 presents the correlation coefficients of the 11 radiomics features included in the model. The Radscore is calculated for each patient based on these features.

**Table 2:** Radiomic feature coefficients were selected using LASSO regression

Radiomics features	Coefficients
logarithm_glcm_MCC_SAT	−0.03428196998005711
square_glrlm_HighGrayLevelRunEmphasis_SAT	-0.019118993971849532
wavelet-LLL_glrlm_GrayLevelNonUniformityNormalized_SMA	-0.008419893905207523
original_glcm_Imc2_SAT	-0.06280275160263817
wavelet-HLL_firstorder_10Percentile_SAT	0.0016932691098839375
wavelet-LHL_glrlm_ShortRunEmphasis_IMAT	-0.04761031463841832
wavelet-LHL_glcm_JointEnergy_SAT	0.016248416539321155
wavelet-LLL_gldm_DependenceVariance_SMA	0.10163205952975779
square_glrlm_ShortRunHighGrayLevelEmphasis_SAT	-0.01554598931696073
wavelet-LHH_glrlm_LongRunEmphasis_SMA	0.03213935897059165
wavelet-LHL_firstorder_Minimum_VAT	0.036032548496018224

**Fig. 4:**
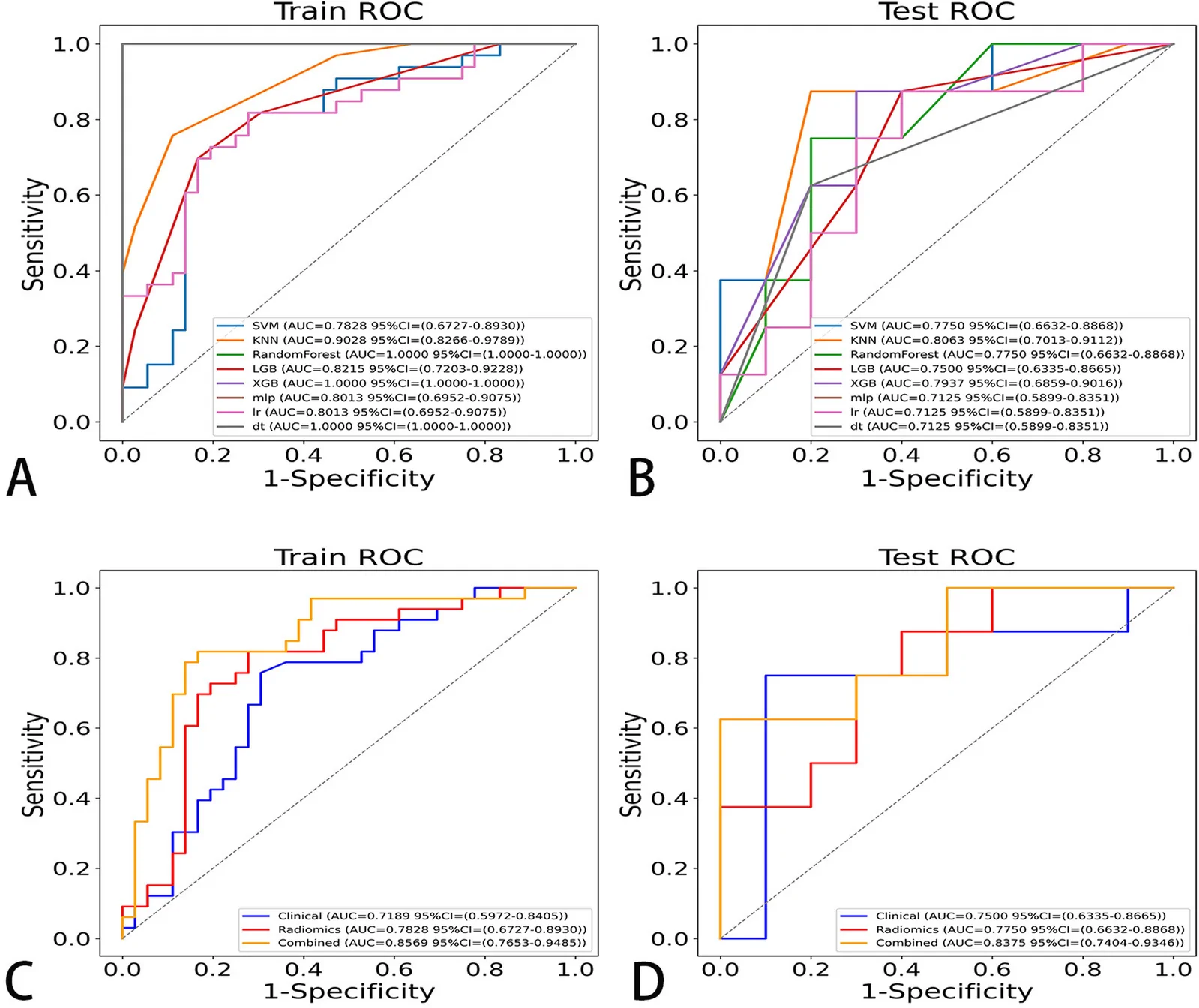
ROC curve. (A) ROC curve for machine learning algorithms in the train cohort; (B) and test cohort; (C) Clinical, Radiomics and Combined model in the train cohort and (D) test cohort

Several machine learning algorithms were employed in the model construction, including Random Forest(RF), Logistic Regression, eXtreme Gradient Boosting (XGBoost), Support Vector Machines (SVM), Light Gradient Boosting Machine (LightGBM), and K-Nearest Neighbors (KNN). We present the optimized hyperparameter configurations for the final models in Supplementary Table 2. The Table 3 presents the performance metrics for each classifier on both the train and test cohort, while Fig. 4 illustrates the ROC for each model in these cohort. Upon comparing the performance metrics, it was found that the differences between most of the models in the train and test cohort were significant. Consequently, the SVM algorithm was identified as the most stable for constructing the radiomics model. The AUC values were 0.782(95% CI:0.673–0.893) for the train cohort and 0.775 (95% CI: 0.663–0.887) for the test cohort, with accuracy rates of 0.768 and 0.722, respectively.

**Table 3:** Model performance in train cohort and test cohort

Model		ACC	AUC(95%CI)	Sensitivity	Specificity
SVM	Train cohort	0.7681	0.7828(0.673–0.893)	0.7273	0.7273
	Test cohort	0.7222	0.7750(0.663–0.887)	0.7500	0.7000
KNN	Train cohort	0.8261	0.9028(0.827–0.979)	0.7576	0.8889
	Test cohort	0.8333	0.8063(0.701–0.911)	0.8750	0.8000
RF	Train cohort	1	1(1–1)	1	1
	Test cohort	0.7222	0.8187(0.717–0.921)	0.6250	0.8000
LGB	Train cohort	0.7681	0.8215(0.720–0.923)	0.6970	0.8333
	Test cohort	0.6667	0.7500(0.634–0.867)	0.6250	0.7000
XGB	Train cohort	0.9855	1(1–1)	0.9697	1
	Test cohort	0.7222	0.7937(0.686–0.902)	0.6250	0.8000
LR	Train cohort	0.7536	0.8013(0.695–0.901)	0.6970	0.8056
	Test cohort	0.7222	0.7125(0.590–0.835)	0.7500	0.7000

*SVM* Support Vector Machine, *RF *Random Forest, *LR *Logistic Regression, *XGB *eXtreme Gradient Boosting, *LGB *Light Gradient Boosting Machine

### Combined model

The Table 4 presents results of univariate Cox regression analyses indicated that the included clinical baseline variables were not statistically significant (*p* > 0.05). This finding is consistent with prior studies, which have identified adipose tissue as a strong prognostic factor in the context of synchronous radiotherapy. TATI was employed for building a clinical model and integrated into the combined model.The Table 5 presents the performance metrics of the combined model, while the Fig. 4 illustrates its ROC. The model incorporating TATI achieved an AUC of 0.857 (95% CI: 0.765–0.949) and 0.834 (95% CI: 0.740–0.935) for the train and test cohort, respectively. The accuracry for these cohort were 0.739 and 0.722, respectively. While the inclusion of TATI has effectively enhanced the AUC of the model, its impact on other performance metrics remains limited.

**Table 4:** Univariate Cox regression in the train cohort

Characteristics	Univariate	
	HR, (95% CI)	*P*
Gender	1.28(0.58–2.84)	0.535
Age	1.01(0.98–1.04)	0.578
ECOG	1(0.52–1.94)	0.989
Path	0.54(0.26–1.1)	0.089
Stage	0.62(0.27–1.42)	0.256
BMI	1.08(0.98–1.19)	0.109
SAT	1(0.99–1.01)	0.735
VAT	1(1-1.01)	0.107
IMAT	1.02(0.97–1.07)	0.402
SMA	1(0.99–1.01)	0.802
SMD	0.97(0.93-1)	0.082
VSR	1.9(0.96–3.77)	0.065
SFI	1(0.98–1.02)	0.802
VFI	1.01(1-1.03)	0.102
IFI	1.06(0.93–1.21)	0.356
SMI	1(0.97–1.04)	0.807
SMG	1(1–1)	0.275
TFA	1(1-1.01)	0.24
TATI	1.01(1-1.02)	0.255
Haemoglobin	1.01(0.99–1.04)	0.288
Neutrophil	1(0.84–1.19)	0.998
Lymphocyte	1.14(0.69–1.89)	0.616
Platelet	1(1–1)	0.911
Transfer	0.61(0.31–1.23)	0.167

*SMG* Skeletal Muscle Gauge, *TFA *total abdominal fat area, *TAFI *Total Fat Tissue Index, *SAT *Subcutaneous adipose tissue, *VAT *Visceral adipose tissue, *IMAT *Intramuscular adipose tissue, *SMD *Skeletal muscle density, *VSR* Visceral/subcutaneous fat ratio, *SFI *Subcutaneous Fat Index, *VFI* Visceral Fat Index, *SMI* Skeletal Muscle Index, *LUCS* Lung squamous cell carcinoma

**Table 5:** Radiomics model and Combined model performance

Model		ACC	AUC(95%CI)	Sensitivity	Specificity
Clinical	Train cohort	0.65	0.719(0.597–0.841)	0.55	0.75
	Test cohort	0.65	0.75(0.634–0.867)	0.6	0.7
Radiomics	Train cohort	0.7681	0.7828(0.673–0.893)	0.7273	0.7273
	Test cohort	0.7222	0.7750(0.663–0.887)	0.75	0.7
Combined	Train cohort	0.739	0.8569(0.765–0.949)	0.8182	0.6666
	Test cohort	0.7222	0.8375(0.740–0.935)	0.628	0.8

### DCA

**Fig. 5:**
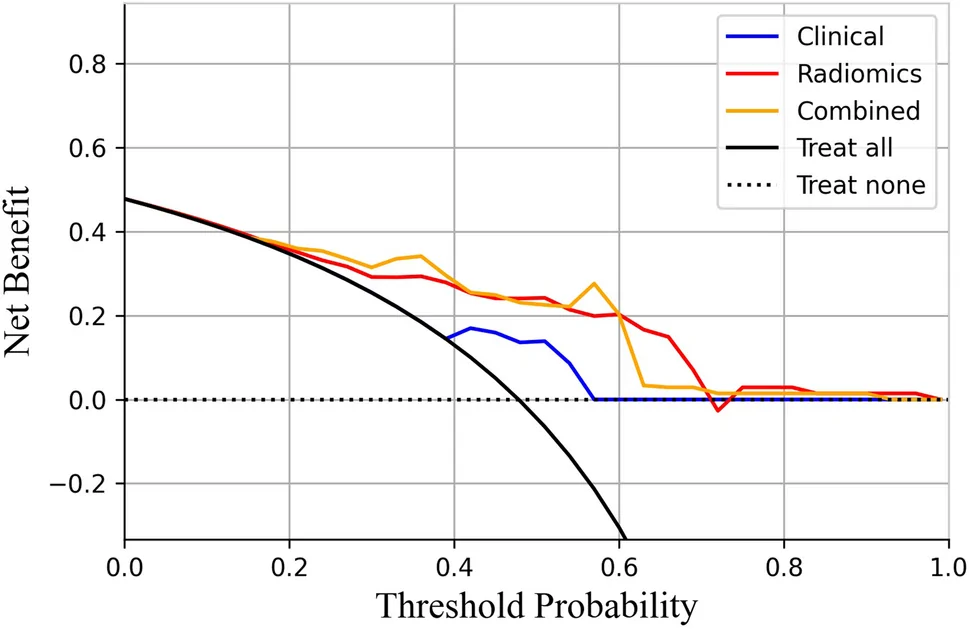
DCA cures

Figure 5 presents the Decision Curve Analysis (DCA) comparing the radiomics, clinical, and combined models. The results show that both the combined and radiomics models consistently yielded higher overall net benefits than the clinical model across most threshold probabilities.

### Relationships between prediction models and PFS

**Fig. 6:**
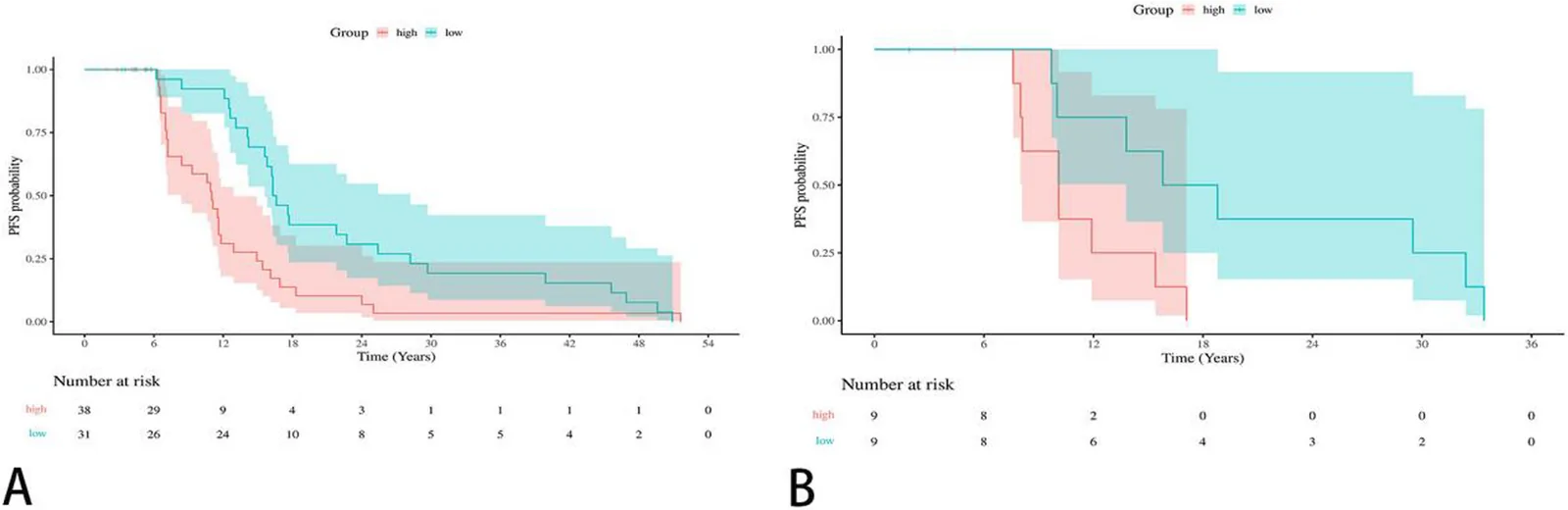
Applying the Combined model, PFS curves for patients with the high risk group and low risk group in the train(A) and test (B)cohort

The Kaplan-Meier survival curves are shown in Fig. 6. In both the train cohort (Fig. 6a, *p* = 0.003) and the test cohort (Fig. 6b, *p* = 0.023), progression-free survival (PFS) was significantly lower for high-risk patients compared to low-risk patients, thereby confirming the high prognostic accuracy of the model.

## Discussion

In this study, radiomics features of skeletal muscle and adipose tissue were extracted to develop a radiomics model for predicting the prognosis of NSCLC patients treated with First-Line Immunochemotherapy and Concurrent Radiotherapy. The SVM-based prediction model, created using five machine learning algorithms, demonstrated superior predictive performance. Additionally, the combination of TATI and radiomics features showed excellent discriminative power, with an AUC of 0.86 for the train cohort and 0.84 for the test cohort. This model can effectively identify advanced NSCLC patients at high risk during simultaneous radiotherapy, facilitating timely clinical intervention and improving patient prognosis. By assisting in cancer patient management, the model enables the development of personalized treatment plans. It can also predict high-risk patients, enhance monitoring, prevent functional deterioration, and improve treatment outcomes, survival rates, and life expectancy. Furthermore, the model can help allocate appropriate medical resources based on patient risk, improving care efficiency, quality, and preventing medical misuse.

Previous studies have primarily focused on the clinical application of radiomics in lung cancer, using imaging biomarkers for diagnosis, staging, prognosis, and the prediction of tumor response. In lung nodule diagnosis, CT scans at lower radiation doses are used to differentiate between benign and malignant nodules and to determine tumor staging [19, 20]. In terms of prognosis, researchers have utilized PET/CT and CT-based radiomics features to predict outcomes in NSCLC patients [21]. For treatment response, established criteria for assessing solid tumor responses are applied to predict whether a patient will respond to radiotherapy [22, 23]. Additionally, the emerging field of radiogenomics combines imaging genomics with gene sequencing to predict tumor cell gene mutations, highlighting the non-invasive nature of imaging genomics as a valuable tool in diagnosis, treatment, and prognosis across various diseases [24–26].

Adipose tissue, particularly visceral fat, is an active endocrine organ secreting adipokines and cytokines that can create a chronic, low-grade pro-inflammatory state and an immunosuppressive tumor microenvironment, potentially dampening the efficacy of immunotherapy [27, 28]. The textural heterogeneity in fat captured by radiomics may be a surrogate for this dysfunctional metabolic activity [29]. Similarly, reduced skeletal muscle density and altered texture may reflect myosteatosis and cachexia, processes driven by systemic inflammation and tumor-derived factors that lead to functional decline and treatment intolerance [30]. In our study, Lasso regression identified a signature of 11 prognostic radiomics features: eight derived from adipose tissue (SAT, VAT) and three from skeletal muscle (SMA, IMAT).The specific profile of selected features offers a compelling, albeit preliminary, biological narrative. The predominance of texture-based features (GLCM, GLRLM, GLDM) from adipose tissue—such as logarithm_glcm_MCC_SAT and wavelet-LHL_glcm_JointEnergy_SAT— suggests that the spatial heterogeneity and architectural disorder of fat deposits are key prognostic factors. In oncology, adipose tissue heterogeneity is increasingly linked to a dysfunctional, pro-inflammatory adipokinesecretome that may foster an immunosuppressive tumor microenvironment, potentially impairing response to immunotherapy [31].

Conversely, the skeletal muscle signature, including features like wavelet-LLL_glrlm_GrayLevelNonUniformityNormalized_SMA, points to the importance of muscle tissue uniformity. Lower uniformity (higher non-uniformity) may reflect myosteatosis or early sarcopenic changes, indicative of systemic metabolic dysregulation and reduced physiological reserve.Notably, the inclusion of first-order features likes wavelet-HLL_firstorder_10Percentile_SAT and wavelet-LHL_firstorder_Minimum_VAT highlights that simple intensity distribution properties (e.g., the prevalence of very low-density fat pixels) also carry prognostic information, possibly correlating with lipid composition or edema [32].Our findings that adiposity and muscle radiomics predict survival are biologically plausible. Thus, our model may encapsulate imaging biomarkers of these deleterious host-tumor interactions.

Cancer-associated cachexia (CAC), body composition, nutrition, and inflammatory status all have significant prognostic and predictive value in terms of survival and treatment-related toxicity [33]. In a multicenter study conducted by Trestini, alterations in SAT density were shown to be associated with prognosis in NSCLC patients undergoing immunotherapy [34]. Further subgroup analyses by Chaunzwa. revealed that sarcopenia was not significantly correlated with prognosis in NSCLC [35]. Additionally, an increased adiposity index was strongly linked to improved survival outcomes [36]. Moreover, body composition imaging was used by Soh to develop a prognostic model for predicting patient survival, achieving an accuracy of 78%. This model outperformed traditional body composition analyses based on reporting indices [37].

The combined model proposed in this study (AUC: 0.85) demonstrates comparable predictive performance to recent tumour-based radiomics studies. For instance, Janzen et al. achieved a similar AUC (0.85) when using tumour radiomics to predict immunotherapy outcomes in advanced NSCLC [38]; in ovarian and cervical cancer prognosis prediction, tumour radiomics models yielded AUC of 0.78 and 0.72 respectively [39, 40]. Crucially, however, these high-value predictive insights derive from entirely distinct biological foundations. Traditional radiomics focuses on tumour-specific heterogeneity, whereas this study systematically demonstrates for the first time that texture features extracted from host adipose and muscle tissue provide comparable independent prognostic information. Our findings provide robust radiomic biomarker evidence for this approach.

In the present study, baseline data for NSCLC patients prior to synchronous radiotherapy did not reach statistical significance. As a result, we incorporated TATI into our combined model to evaluate the potential of adipose tissue as a prognostic factor. Compared to body composition radiomics alone, the combined model demonstrated an AUC ranging from 0.78 to 0.86 in the train cohort and 0.78 to 0.84 in the test cohort. Furthermore, the combined model provided a greater net benefit, as indicated by the DCA, suggesting that TATI could serve as a valuable predictor of prognosis.This model robustly identifies ‘high-risk’ and ‘low-risk’ patient subgroups. This binary output directly informs clinical decision-making. For instance, patients categorised as high-risk may receive prioritised optimisation of personalised management, whilst identifying relapse risk can guide interventions including physical training, nutritional supplementation and targeted therapy to improve prognosis [41–43].Conversely, low-risk patients may undergo standard follow-up management, optimising resource allocation. Notably, this tool utilises existing CT scan data without requiring additional examinations, enhancing clinical judgement through objectively quantified risk assessment.

This study has several limitations.First, the retrospective, single-center design inherently carries a high risk of selection bias. Our patient cohort may not be fully representative of the broader advanced NSCLC population, as it reflects the specific diagnostic and treatment protocols, as well as the demographic characteristics, of a single institution. This limits the external validity of our model. Second, the relatively small sample size substantially constrains the statistical power and increases the risk of model overfitting. While internal validation was attempted, a model developed on a limited dataset may identify spurious or cohort-specific patterns that do not hold true in larger, more diverse populations. This directly questions the stability and reliability of the identified radiomics features and the integrated TATI model. Third, image segmentation was performed manually, a time-consuming process that limits the clinical applicability of the method in larger cohorts. Additionally, there is potential for bias in the description of the ROI, as inconsistencies in its delineation by different imaging physicians may lead to discrepancies in feature extraction [44]. These discrepancies could be mitigated by adopting an automated segmentation approach, which would improve the efficiency of the process and ensure the stability and consistency of the analyses.

In the future, to advance the application of body composition in lung cancer prognostic models, external validation of the model within large-scale, multicentre prospective cohorts may be required to confirm its generalisability and rigorously assess its performance in real-world settings. Building upon this foundation, subsequent research should focus on optimising algorithms and enhancing predictive performance. This could involve integrating deep learning, incorporating additional known prognostic factors, and leveraging multi-omics data such as genomics and proteomics to generate more robust evidence.

## Conclusion

Baseline body composition and radiomics features in advanced NSCLC patients may have potential as prognostic indicators. Our preliminary study suggests that integrating these features with the TATI machine learning model shows promise in predicting patient prognosis from baseline CT images. If validated in larger studies, this approach could contribute to precision medicine and aid clinical decision-making for NSCLC patients.

## Supplementary Information


Supplementary Material 1.


## Data Availability

Data generated or analyzed during the study are available from the corresponding author by request.

## Abbreviations

NSCLC: Non-small cell lung cancer
CRT: concurrent radiotherapy
BMI: While body mass index
PFS: progression-free survival
CT: computed tomography
SMA: skeletal muscle cross-sectional area
SAT: subcutaneous fat area
VAT: visceral fat area
IMAT: intramuscular fat area
ML: machine learning
ROI: regions of interest
RF: Random Forest
LR: Logistic Regression
XGBoost: eXtreme Gradient Boosting
SVM: Support Vector Machines
LightGBM: Light Gradient Boosting Machine
KNN: K-Nearest Neighbors
