# Supplementary material for: Body composition radiomics integrated with machine learning to predict prognosis in advanced NSCLC treated with first-line immunochemotherapy and concurrent radiotherapy
Source: BMC Pulm Med. 2026 Jun 19;26:385. doi: 10.1186/s12890-026-04405-w (PMC13527977; doi:10.1186/s12890-026-04405-w)
Supplement: Supplementary file 1 [file 12890_2026_4405_MOESM1_ESM.docx]

**Supplementary Material**

**Supplementary Table** **1** Hyperparameter Search Space for Machine Learning Classifiers

| Classifier | Key Hyperparameters (Search Range) |
| --- | --- |
| LGB | n_estimators: [100, 200]; num_leaves: [31, 50]; learning_rate: [0.01, 0.1] |
| XGB | n_estimators: [100, 200]; max_depth: [3, 6]; learning_rate: [0.01, 0.1] |
| RF | n_estimators: [100, 200, 300]; max_depth: [5, 10, None] |
| LR | C: [0.01, 0.1, 1, 10]; penalty: ['l1', 'l2'] |
| KNN | n_neighbors: [3, 5, 7]; weights: ['uniform', 'distance'] |
| SVM | C: [0.1, 1, 10]; gamma: ['scale', 0.01, 0.1]; kernel: ['rbf'] |

LGB, LightGBM; MLP, Multilayer Perceptron; XGB, eXtreme Gradient Boosting;

RF,Random forest; LR, Logistic Regression; DT,Decision Tree.

**Supplementary Table** **2** The hyperparameters used in model training.

| Model | Parameters | Values |
| --- | --- | --- |
| LGB | learning_rate | 0.01 |
|  | estimators  num_leaves | 100 |
|  |  | 31 |
| XGB | learning_rate | 0.1 |
|  | max_depth  n_estimators | 3  100 |
| RF | max_depth | 5 |
|  | n_estimators | 100 |
| LR | C  penalty | 0.1  'l1' |
| KNN | weights  n_neighbors | 'uniform'  3 |
| SVM | C | 1 |
|  | gamma | 0.01 |
|  | kernel | 'rbf' |

LGB, LightGBM; MLP, Multilayer Perceptron; XGB, eXtreme Gradient Boosting;

RF,Random forest; LR, Logistic Regression; DT,Decision Tree.
